# The Clinical Registry of Childhood Asthma (CRCA) Elucidating Early-Life Asthma: Cross-Sectional Analysis of a Prospective, Longitudinal, and Digitally Enhanced Real-World Cohort

**DOI:** 10.2196/78693

**Published:** 2025-10-30

**Authors:** Juan Li, Luo Ren, Jiao Liu, Yuyi Tang, Run Wang, Peixin Yang, Jing Zhao, Xiao Chen, Zheng Xiang, Wen Zhong, Na Zang, Dapeng Chen, Heping Fang, Enmei Liu

**Affiliations:** 1 Department of Respiratory Medicine, Children’s Hospital of Chongqing Medical University, National Clinical Research Center for Child Health and Disorders Ministry of Education Key Laboratory of Child Development and Disorders, Chongqing Key Laboratory of Child Rare Diseases in Infection and Immunity Key Laboratory of Children’s Important Organ Development and Diseases of Chongqing Municipal Health Commission Chongqing China; 2 Children’s Hospital of Chongqing Medical University Pediatric Research Institute Chongqing China; 3 Department of Clinical Laboratory, Children’s Hospital of Chongqing Medical University Chongqing China; 4 Guangzhou National Laboratory Guangzhou China

**Keywords:** asthma, early life, digital health, prospective cohort studies, real-world method, patient selection

## Abstract

**Background:**

Childhood asthma, particularly in early life, is often underdiagnosed and poorly characterized in real-world outpatient settings due to diagnostic challenges and resource constraints. A pragmatic, scientifically rigorous, prospective cohort model is urgently needed.

**Objective:**

We aimed to establish and present a cross-sectional baseline analysis of the Clinical Registry of Childhood Asthma, a prospective, longitudinal, and digitally enhanced cohort in outpatient settings, focusing on the diagnostic spectrum of early-life asthma.

**Methods:**

We established the Clinical Registry of Childhood Asthma cohort and performed a cross-sectional analysis of its baseline data. We launched the cohort in March 2024 as an ongoing study, enrolling children (<18 years) with persistent cough and wheezing from a tertiary pediatric referral center in Southwest China. The study used a real-world design, integrating symptom-driven recruitment with standardized electronic medical records, structured electronic patient-reported outcomes, and systematic biobanking of residual biospecimens. Participants were classified as having confirmed, suspected, or excluded asthma based on cross-sectional baseline data.

**Results:**

From March 2024 to August 2025, we enrolled 396 children (median age 4.7 years) from 2296 outpatient visits (enrollment rate 17.2%). Follow-up rates were 26.7% and 43.3% at first and second timepoints, respectively. A comprehensive biorepository was established with serum, plasma, PBMCs, and other blood cell samples (average coverage 74.0%). Most children (267/396, 67.4%) were under 6 years. Patients were stratified into confirmed (131/396, 33.1%), suspected (179/396, 45.2%), and excluded asthma (86/396, 21.7%). Suspected and excluded cases were significantly younger than confirmed cases (median 4.1/3.9 vs 6.6 years, *P*<.001). Comorbidity profiles differed significantly: allergic rhinitis prevailed in confirmed asthma (77/131, 58.8%), while chronic cough (64/86, 74.4%) and bronchitis (39/86, 45.3%) dominated the excluded group. Type 2 inflammation biomarkers also differed across groups, including aeroallergen sensitization, blood eosinophil count, and fractional exhaled nitric oxide (*P*<.001). Physician-parent diagnostic discordance was most pronounced in suspected asthma (76/134, 56.7%, *P*<.001). Multivariable analyses showed suspected asthma (vs excluded) was associated with respiratory infection as wheezing trigger (odds ratio [OR] 4.41, 95% CI 2.16-9.42, *P*<.001), family history of allergic rhinitis (OR 2.27, 95% CI 1.08-4.99, *P*=.03), and higher blood eosinophil count (OR 1.32 per 100 cells/μL, 95% CI 1.05-1.73, *P*=.02). Confirmed asthma (vs suspected) was associated with older age (OR 1.29 per year, 95% CI 1.14-1.47, *P*<.001), allergic rhinitis (OR 4.06, 95% CI 1.99-8.31, *P*<.001), and aeroallergen sensitization (OR 3.83, 95% CI 1.91-7.66, *P*<.001). Bronchitis was negatively associated with an asthma diagnosis across models (OR 0.30 for suspected vs excluded; OR 0.21 for confirmed vs suspected; OR 0.13 for confirmed vs excluded).

**Conclusions:**

The Clinical Registry of Childhood Asthma establishes a feasible cohort in outpatient settings that captures the diagnostic uncertainty of early-life asthma. It identifies a distinct suspected asthma subgroup and reveals significant patient-clinician diagnostic discordance, providing a valuable resource for improving disease management.

## Introduction

Asthma is a chronic respiratory disease affecting 262 million people worldwide, with children representing a particularly vulnerable population [1]. High prevalence rates have been reported in urban regions of China and India [2]; yet, early-life asthma remains both undermanaged in clinical practice and underrepresented in research [3]. This is due to several challenges, including the fast-paced nature of pediatric outpatient clinics where most children are treated [4], the inherent heterogeneity of asthma [5], and diagnostic difficulties—especially in children under 6 years old [3]. These factors contribute to low rates of diagnostic confirmation and inhaled corticosteroid adherence, exacerbating the overall disease burden [2,6].

To address these issues, the Global Initiative for Asthma (GINA) 2025 report has introduced, for the first time, diagnostic criteria and a definition for suspected asthma in children under 6 years old [3]. Despite this important step, the characteristics of early-life asthma in outpatient settings remain poorly characterized. This gap is particularly critical since the first 5 years of life constitute a key window for allergen sensitization [7], the foremost risk factor for childhood asthma. Therefore, there is an urgent need to establish well-characterized cohorts of young asthmatic children within outpatient settings to improve understanding and management.

To date, 3 primary approaches have been used to study childhood asthma. Large-scale birth cohorts have advanced our understanding of asthma and allergic diseases [8-12] but require substantial resources and yield limited cases due to disease incidence. Small-scale prospective asthma cohorts often include children with established diagnoses (typically over 6 years old) [13-18]; yet, this may restrict exploration of early disease heterogeneity. Retrospective cohorts based on hospital records [19-25] offer real-world insights but are limited by incomplete data and suboptimal biospecimen preservation. Given these limitations, an integrated model that combines prospective and retrospective approaches within routine clinical practice offers a promising alternative.

Emerging strategies enhance the feasibility and rigor of such an integrated model. Real-world evidence methodologies and symptom-driven phenotyping enable early identification of children with asthmatic symptoms, even before formal diagnosis [26-29]. Standardized electronic medical records (EMRs) improve data completeness and accessibility [30,31], while electronic patient-reported outcomes (ePROs) capture symptoms and experiences beyond clinical encounters [32-34]. In addition, residual biospecimens from routine testing can be repurposed for molecular endotyping using high-throughput omics technologies, minimizing invasive procedures [35-38]. These tools not only reduce cohort heterogeneity but also facilitate data harmonization and future integration, mirroring successful efforts in major birth cohorts [12,39].

In this study, we developed the Clinical Registry of Childhood Asthma (CRCA)—an outpatient-based integrated research platform that combines symptom-driven recruitment, real-world evidence collection, standardized EMRs, structured ePROs, and residual biospecimens. This framework supports both the establishment of a dynamically updated biorepository and enables phenotyping of early-life asthma. We aimed to characterize children across confirmed, suspected, and excluded asthma categories and identify factors associated with these diagnostic outcomes.

## Methods

### Study Design

**Figure 1:**
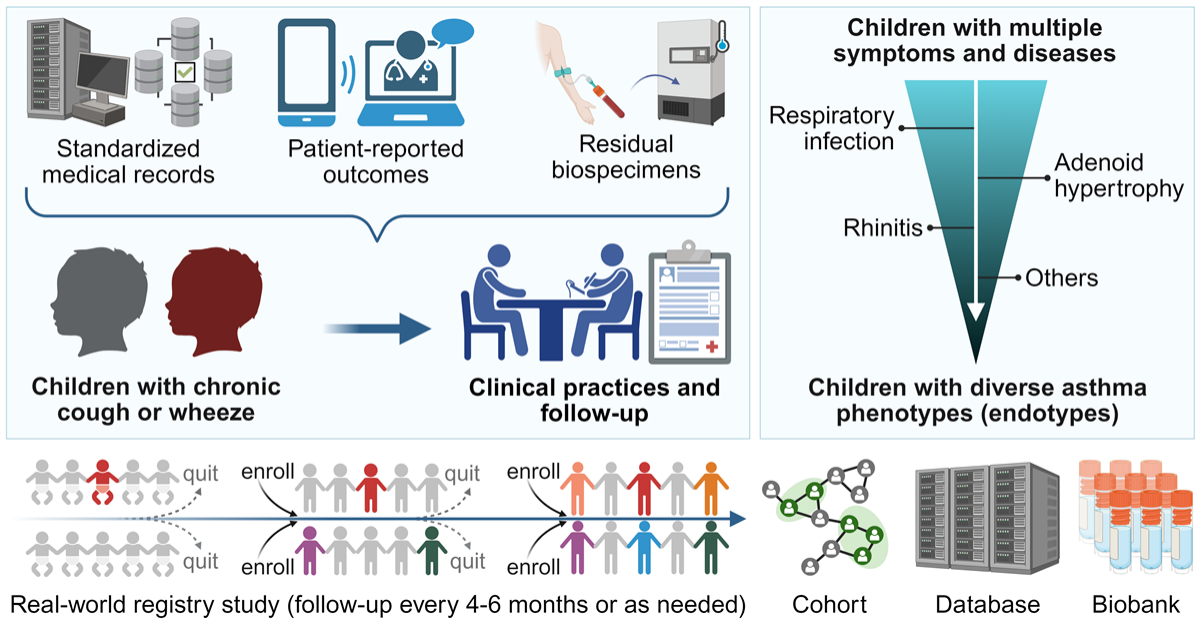
Design and inclusion procedure of the study (created with BioRender.com).

The CRCA is a prospective, longitudinal, digitally enhanced real-world cohort study launched on March 7, 2024, at the Respiratory Clinic of the Children’s Hospital of Chongqing Medical University—a tertiary pediatric referral center in Southwest China. Designed to address the critical gap in well-characterized outpatient cohorts for early-life asthma, the CRCA operates as an open, integrated research framework embedded within routine clinical workflows to enable long-term follow-up (Figure 1). This manuscript presents a cross-sectional analysis of the baseline data from this ongoing cohort.

This study used a symptom-driven recruitment strategy to proactively identify and enroll children during visits [27,29]. While this approach may initially include children with a broader spectrum of wheezing illnesses (“off-target” recruitment) [40] and is subject to real-world follow-up challenges [41-43], it is conceptualized not as a limitation but as a fundamental feature enabling the construction of a dynamic, funnel-shaped biorepository for long-term observational research. Through continuous enrollment, the framework accumulates a diverse population, with data collection occurring at scheduled follow-up intervals, which naturally refines over time into a well-characterized cohort of children with confirmed or suspected asthma, while also preserving longitudinal data on the broader natural history of childhood wheezing.

The term “digitally enhanced” refers to our core strategy of synthesizing two complementary data streams collected overtime: (1) hospital-based EMRs for structured clinical data and (2) smartphone-based ePROs for repeatedly capturing symptom and experience data from caregivers beyond clinical encounters. This dual-stream approach ensures comprehensive and continuous longitudinal data acquisition within a high-volume outpatient setting.

The primary outcomes for this article are the detailed cross-sectional characteristics of children with early-life asthma, stratified by diagnostic status (confirmed, suspected, or excluded). Secondary outcomes include metrics related to the implementation of the CRCA study, including the feasibility (>70% ePRO completion and biospecimens collection rates) and sustainability of recruitment efforts. In-depth analysis of longitudinal outcomes is beyond the scope of this initial report, but it is a core purpose of the ongoing cohort. Reporting follows the STROBE (Strengthening the Reporting of Observational Studies in Epidemiology) guidelines (Multimedia Appendix 1).

### Ethical Considerations

The study protocol was approved by the Ethics Committee of the Children’s Hospital of Chongqing Medical University (approval number 2024-37) and registered with China’s National Medical Research Registration and Recording Information System (registration number MR-50-24-049642). Written informed consent was obtained from all participating parents, with additional assent from children aged ≥8 years. No financial compensation was provided. The study imposed no additional diagnostic procedures or financial burdens beyond standard care. All procedures adhered to the Declaration of Helsinki, and participant confidentiality was maintained.

### Recruitment and Follow-Up

The longitudinal design of CRCA is implemented through a structured recruitment and follow-up protocol. Eligible participants were children under 18 years of age who presented with persistent or recurrent cough, wheezing, or both, lasting at least 1 month. In addition, asthma needed to be suspected by either the referring practitioner or our study pediatricians. Finally, the child’s clinical assessment or a parental request had to indicate the need for blood tests, such as serum-specific IgE (sIgE) testing. Participants who met the inclusion criteria and elected to continue follow-up at the CRCA clinic were enrolled. It is important to note that while the CRCA registry enrolls children across the entire pediatric age range (<18 years) to establish a comprehensive and longitudinal resource, this initial cross-sectional analysis focuses specifically on the early-life period, where diagnostic uncertainty is greatest.

Enrolled participants are followed prospectively every 4-6 months or as clinically indicated, in accordance with GINA-based management [3]. Study exit was defined as either (1) clinical resolution of symptoms or (2) failure to attend follow-up for over one year. However, given the chronic and relapsing nature of asthma, children who returned to the clinic after study exit, whether for exacerbations or routine management, were re-eligible for data collection (Figure 1).

### Definitions of Asthma Diagnosis

Asthma status was classified for all ages as “confirmed” or “suspected” per GINA 2025 [3]. For children under 6 years, a “confirmed asthma” diagnosis required meeting 3 criteria. First, the child must have had recurrent acute wheezing episodes, which could occur with or without interval symptoms. Second, alternative diagnoses must have been excluded. Third, a timely response to asthma treatment was necessary. A “suspected asthma” classification was applied when there was a highly suggestive clinical history that lacked objective confirmation. This lack of confirmation could be due to the infeasibility of testing or an incomplete treatment response. In such cases, alternative diagnoses were also considered unlikely. For individuals ≥6 years, “confirmed asthma” required objective evidence of variable expiratory airflow limitation, demonstrated by positive bronchodilator reversibility, excessive peak expiratory flow variability, or a positive bronchial challenge test. “Suspected asthma” was defined by characteristic symptoms in the absence of confirmatory test results, whether due to testing infeasibility or negative or equivocal results despite a suggestive history.

### Standard EMRs

**Figure 2:**
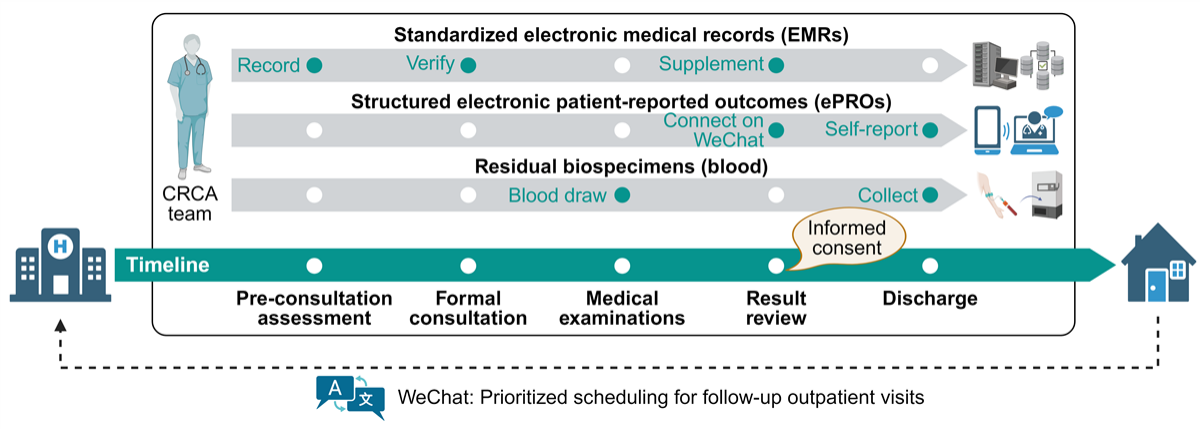
Timeline and workflow of the study (created with BioRender.com). EMR: electronic medical record; ePRO: electronic patient-reported outcome.

The CRCA study implemented a standardized EMR template to ensure consistent data collection. The baseline information module captured comprehensive demographic characteristics, including the child’s full name, date of birth, residential address, contact information, and body weight. The clinical assessment module systematically recorded medical data across multiple domains. These included respiratory symptoms, concomitant conditions, and current medications. It also captured treatment responses, physical examination findings, and laboratory results such as complete blood counts and allergen sensitization profiles (serum sIgE tests). Additionally, objective measures like lung function and fractional exhaled nitric oxide (FeNO) were documented. This module also documented complete medical histories, encompassing perinatal events, personal atopy history, previous severe pneumonia episodes, and mechanical ventilation history. Data were initially collected by research staff during preconsultation assessments, then verified and supplemented during formal consultations and test result reviews (Figure 2).

### ePROs

Parents (primarily mothers) of eligible participants were invited to connect with the official WeChat account of the CRCA study. This account fulfilled 3 essential functions: establishing a direct pediatrician-parent communication channel for clinical inquiries, serving as the platform for ePROs collection, and enabling prioritized scheduling of follow-up outpatient visits.

At enrollment, parents received a voluntary web-based questionnaire administered through WeChat, adapted from our previous and related studies to capture multidimensional quantitative data [44-46]. This instrument collected information across key domains, including sibling composition, family structure, familial atopy history, residential characteristics, pet ownership, indoor air quality, hygiene practices, household chemical usage patterns, health status, asthma risk factors, pharmacological treatment history, and symptom control metrics using age-appropriate Asthma Control Test (ACT) or Childhood Asthma Control Test (C-ACT). Each questionnaire included a self-rated response quality assessment item to ensure high-quality data collection. The primary analytical objective for this comprehensive environmental exposure data is to determine its associations with key asthma-related outcomes. These outcomes include diagnostic status, symptom control, and exacerbation frequency. Our planned analysis will use multivariate statistical models to quantify these exposure-outcome relationships while adjusting for potential confounders, including demographic and familial risk factors. For longitudinal monitoring, abbreviated follow-up questionnaires collected core demographics alongside age-appropriate ACT or C-ACT measures at each scheduled follow-up time point.

### Residual Biospecimens

Residual biospecimens (primarily blood) were collected by CRCA staff through the Clinical Laboratory Department, typically during the afternoon following blood collection. Samples were processed to isolate plasma, serum, peripheral blood mononuclear cells (PBMCs), and other blood cells. Serum and plasma were aliquoted at 200 μL per tube, PBMCs at 500 μL per tube, and other blood cells were stored in one tube per participant. All aliquoted samples were cryopreserved. PBMCs underwent programmed freezing. They were first stored at –80° C for over 12 hours before their final transfer to liquid nitrogen. All samples were ultimately categorized and stored under standardized conditions (–80° C freezers or liquid nitrogen) within our institution’s Biobank Center. All associated metadata were tracked electronically. This process followed protocols established by the Western China Birth Cohort study [37]. Residual blood specimens were not used in the present analysis to avoid purposeless consumption. However, the scientific value of these biospecimens is well-established. This is demonstrated by prior multiomics applications in Mycoplasma pneumoniae pneumonia research, which used identical specimen collection protocols [38].

### Quality Control and Data Security

The CRCA working group comprised trained pediatricians and clinical researchers who met monthly to review and optimize operations. All data were stored locally under anonymized identifiers. Access to data and biospecimens required approval from the principal investigator under stringent security protocols.

A dedicated team managed the EMR data extraction. Initial collection and entry were performed by Juan L and HF, and are now managed by PY and JZ. All EMR data were reviewed and verified by EL. ePROs followed rigorous methodological standards from our previous work [47,48]. Biospecimen handling adhered to stringent Chinese National Standards for human blood biobanking; our group contributed to drafting “Human blood sample biobanking—Part 1: General specifications” (Plan #20253246-T-469) and “Part 2: Peripheral blood mononuclear cells” (Plan #20253247-T-469), publicly accessible on China’s National Public Service Platform for Standards Information. This ensures long-term sample quality and readiness for integration with clinical outcomes.

### Statistical Analyses

Data analysis and plotting were performed using R 4.4.1, GraphPad Prism 9, and the BioRender website. Categorical variables are presented as frequencies (percentages) while continuous variables are shown as medians (IQRs) following normality assessment. Missing data were not imputed for 2 primary reasons. First, missingness in lung function and FeNO measurements was deemed not missing at random, as it was highly correlated with disease severity and clinical progression. Second, missing questionnaire data typically involved entire unreturned forms; imputation of such large-scale missingness was considered to introduce unacceptable bias. Therefore, all analyses were performed on complete cases.

Participants were stratified by diagnostic category (confirmed, suspected, and excluded asthma). Comparative analyses applied chi-square tests for categorical variables, and Kruskal-Wallis tests with Bonferroni correction for multiple comparisons. A 2-sided *P*<.05 was considered statistically significant. For multivariable analyses, three logistic regression models were constructed: (1) Model 1 compared suspected versus excluded asthma; (2) Model 2 compared confirmed versus suspected asthma; and (3) Model 3 compared confirmed versus excluded asthma. We used a tiered variable selection strategy to enhance robustness. Univariate screening was conducted at three thresholds (*P*<.30, *P*<.20, and *P*<.10). The model using the strictest threshold (*P*<.10) served as the primary final model, while those using more lenient thresholds (*P*<.30 and *P*<.20) were used in sensitivity analyses to evaluate the stability of core associations. Within each tier, forward stepwise likelihood ratio selection was applied, with age and sex retained in all models irrespective of significance. All models are based on the cross-sectional baseline diagnosis. The stability of these diagnoses overtime and their evolution will be the subject of future longitudinal analyses of this cohort.

## Results

### Implementation and Feasibility of the CRCA Study

**Figure 3:**
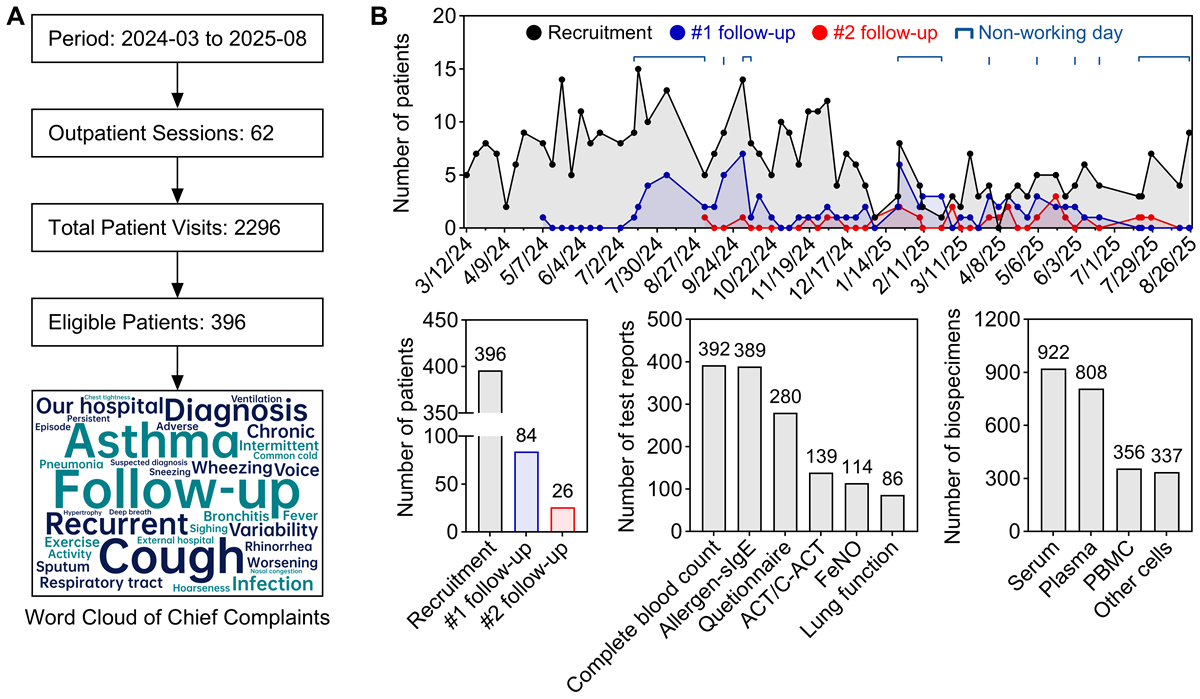
Implementation and feasibility of the CRCA study. (A) Enrollment workflow and chief complaint analysis; (B) Recruitment and follow-up timeline, EMRs collection, and biorepository construction. ACT: Asthma Control Test; C-ACT: Childhood Asthma Control Test.

Between March 2024 and August 2025 (Figure 3A), we organized 62 weekly outpatient sessions, evaluating an average of 37 patients per session (total 2296 visits; mean consultation time: 7.3 minutes). From this population, 396 eligible patients were enrolled (enrollment rate: 17.2%). Initial and second follow-up assessments were completed by 26.7% (84/315) of eligible patients at the first time point and 43.3% (26/60) of those eligible for the second follow-up, respectively (Figure 3B). Temporal patterns indicated that follow-up completion rates peaked during school vacations and national holidays, reaching 1.4 times the baseline. These results demonstrate the feasibility of implementing a comprehensive research framework within the constraints of a high-throughput pediatric clinic, and underscore the need for flexible, family-centered follow-up strategies in real-world studies.

The integrated data and biospecimen repository constitutes a core output of the CRCA study. All enrolled children have standardized EMRs, which include 392 complete blood count reports, 389 allergen-sIgE reports, 86 lung function tests, 114 FeNO measurements, 280 parental questionnaires, and 139 ACT or C-ACT assessments. Furthermore, the biorepository has accumulated 2423 residual biospecimens (Figure 3B), with coverage rates as follows: serum samples from 357 (90.2%) children, plasma from 317 (80.1%), PBMCs from 171 (43.2%), and other blood cell samples from 327 (82.6%), yielding an average coverage of 74.0% across these specimen types. The rate of hemolysis occurrence was 4.0% (16/396). These coverage rates meet the prespecified feasibility indicators for the biorepository component of the study.

### Clinical Characteristics and Diagnostic Patterns

**Figure 4:**
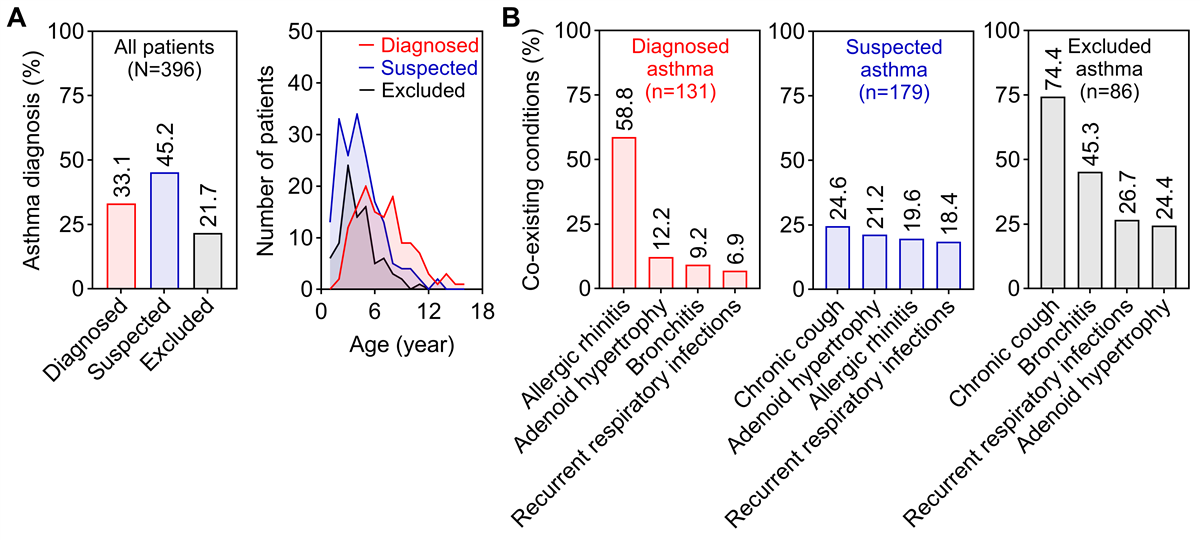
Age and diagnosis distribution of the patients. (A) Characteristics of age and diagnosis; (B) Patterns of coexisting conditions across asthma diagnostic groups.

The CRCA successfully captured a predominantly young cohort, with 267 (267/396, 67.4%) children younger than 6 years. Patients were classified into 3 diagnostic categories: confirmed asthma (131/396, 33.1%), suspected asthma (179/396, 45.2%), and excluded asthma (86/396, 21.7%; Figure 4A). Comorbidity profiles differed markedly among groups (Figure 4B). Allergic rhinitis was most prevalent in the confirmed asthma group (77/131, 58.8%). Children in the excluded asthma group were most frequently diagnosed with chronic cough (64/86, 74.4%) or bronchitis (39/86, 45.3%). In contrast, the suspected asthma group showed a lower prevalence of additional diagnoses, suggesting a clinically distinct, “pure” early wheezing phenotype that is difficult to classify using current criteria.

As shown in Table 1, children with suspected or excluded asthma were significantly younger than those with confirmed asthma (*P*<.001), indicating that the under-6 age group largely comprises uncertain or excluded cases.

**Table 1:** Characteristics of the patients according to diagnosis groups.

		Overall (n=396)	Diagnosis groups			*P* values	
			Confirmed	Suspected	Excluded		
**Age (year), median (IQR)**		4.7 (3.7)	6.6 (3.8)^a,b^	4.1 (3.3)^c^	3.9 (2.4)^a^	<.001	
**Sex, n (%)**							.38
	Male	237 (59.8)	82 (62.6)	109 (60.9)	46 (53.5)		
	Female	159 (40.2)	49 (37.4)	70 (39.1)	40 (46.5)		
**Province, n (%)**							.26
	Sichuan	170 (42.9)	55 (42.0)	74 (41.3)	41 (47.7)		
	Chongqing	168 (42.4)	61 (46.6)	75 (41.9)	32 (37.2)		
	Guizhou	44 (11.1)	13 (9.9)	24 (13.4)	7 (8.1)		
	Others	14 (3.5)	2 (1.5)	6 (3.4)	6 (7.0)		
**Term or preterm birth, n (%)**							.75
	Term	368 (92.9)	120 (91.6)	168 (93.9)	80 (93.0)		
	Preterm	28 (7.1)	11 (8.4)	11 (6.1)	6 (7.0)		
**Personal history of eczema, n (%)**							.01
	Yes	230 (58.1)	79 (60.3)	113 (63.1)^b^	38 (44.2)^a^		
	No	166 (41.9)	52 (39.7)	66 (36.9)	48 (55.8)		
**Personal history of severe pneumonia or invasive mechanical ventilation, n (%)**							.95
	Yes	26 (6.6)	9 (6.9)	11 (6.1)	6 (7.0)		
	No	370 (93.4)	122 (93.1)	168 (93.9)	80 (93.0)		
**First visit for this problem, n (%)**							<.001
	Yes	146 (36.9)	14 (10.7)^a,b^	84 (46.9)^c^	48 (55.8)^c^		
	No	250 (63.1)	117 (89.3)	95 (53.1)	38 (44.2)		
**Completion of initial follow-up, n=315, n (%)**							<.001
	Yes	84 (73.3)	48 (45.3)^b^	28 (20.3)^c^	8 (11.3)^c^		
	No	231 (26.7)	58 (54.7)	110 (79.7)	63 (88.7)		
**Coexisting conditions (nonexclusive), n (%)**							
	Allergic rhinitis	119 (30.1)	77 (58.8)^a,b^	35 (19.6)^c^	7 (8.1)^c^	<.001	
	Chronic cough	113 (28.5)	5 (3.8)^a,b^	44 (24.6)^b,c^	64 (74.4)^b,c^	<.001	
	Bronchitis	79 (19.9)	12 (9.2)^b^	28 (15.6)^b^	39 (45.3)^b,c^	<.001	
	Adenoid hypertrophy	75 (18.9)	16 (12.2)	38 (21.2)	21 (24.4)	.046	
	Recurrent respiratory infections	65 (16.4)	9 (6.9)^b^	33 (18.4)^c^	23 (26.7)^c^	<.001	
	Pneumonia	21 (5.3)	4 (3.1)	10 (5.6)	7 (8.1)	.26	
**Positive rate of serum allergen-specific IgE (nonexclusive), n=390, n (%)**							
	Aeroallergens	144 (36. %)	85 (66.9)^a,b^	44 (24.7)^c^	15 (17.6)^c^	<.001	
	Food allergens	110 (28.2)	42 (33.1)	45 (25.3)	23 (27.1)	.32	
**BEC^d^ (cells/μL), n=392, median (IQR)**		250 (298)	360 (423)^a,b^	250 (250)^b,c^	170 (145)^b,c^	<.001	
**FEV_1_^e^ %Pred (%), n=83, median (IQR)**		100.1 (19.6)	97.3 (14.9)	102.4 (26.5)	109.3 (20.3)	.25	
**FeNO^f^ (ppb), n=114, median (IQR)**		16.5 (17.0)	22.0 (23.0)^a^	12.0 (7.0)^c^	11.0 (11.8)	<.001	

^a^Adjusted *P* value <.05 compared with the suspected asthma group.

^b^Adjust *P* value <.05 compared with the excluded asthma group.

^c^Adjusted *P* value <.05 compared with the confirmed asthma group.

^d^BEC: blood eosinophil count.

^e^FEV1%Pred: Forced Expiratory Volume in 1 second as a percentage of Predicted.

^f^FeNO: fractional exhaled nitric oxide.

These groups also included more first-time visitors (*P*<.001), highlighting the initial visit as a critical yet uncertain diagnostic window. Significant differences in comorbidity patterns were observed across diagnostic categories. Among children with a previous asthma treatment history (n=250), specialist evaluation confirmed asthma in only 46.8% (117/250) and definitively excluded it in 15.2% (38/250), underscoring substantial community-level under- and overdiagnosis. As expected, objective measures of type 2 inflammation and lung function—including aeroallergen sensitization, blood eosinophil counts, and FeNO—differed significantly across groups (*P*<.001), supporting the clinical validity of the diagnostic stratification.

### Self-Reported Characteristics and Patient-Physician Discordance

The study achieved a 70.7% (280/396) questionnaire completion rate, with no significant demographic or diagnostic differences between responders and nonresponders. A central finding was a pronounced discordance between physician diagnoses and parental disease perception (*P*<.001; Table 2). Only 75.6% (68/90) of parents of children with physician-confirmed asthma acknowledged the diagnosis, while merely 57.1% (32/56) of those whose children were ruled out for asthma accepted this conclusion. This difference in diagnostic consistency was most pronounced in the suspected asthma group (discordance rate: 76/134, 56.7%), highlighting a major communication gap in clinical management.

Beyond this discordance, analysis of clinical and environmental factors revealed several patterns more common in the confirmed and suspected asthma groups. Respiratory infections were the predominant wheezing trigger in these groups (62/90, 68.9% and 84/134, 62.7%, respectively). Similarly, seasonal exacerbations in spring and winter were notably more frequent. Inhaled medication use was reported in 60.3% (135/224) of these combined groups, compared to 52.5% (147/280) in the entire cohort. Despite this, 22.3% of patients had uncontrolled symptoms (ACT or C-ACT < 20), indicating a substantial need for improved treatment adherence and effectiveness in real-world practice.

**Table 2:** Self-reported characteristics of the patient according to diagnosis groups.

			Overall (n=280)		Diagnosis groups						*P* values	
					Confirmed		Suspected		Excluded			
**Patient-reported asthma, n (%)**												<.001
	Confirmed	122 (43.6)		68 (75.6)^a,b^		50 (37.3)^b,c^		4 (7.1)^b,c^				
	Suspected	96 (34.3)		18 (20.0)		58 (43.3)		20 (35.7)				
	Excluded	62 (22.1)		4 (4.4)		26 (19.4)		32 (57.1)				
**Patient-physician discordance, n (%)**			122 (43.6)		22 (24.4)^a^		76 (56.7)^c^		24 (42.9)		<.001	
**Family history of related diseases in first-degree relatives (nonexclusive), n (%)**												
	Asthma	45 (16.1)		17 (18.9)		24 (17.9)		4 (7.1)		.12		
	Allergic rhinitis	104 (37.1)		33 (36.7)		56 (41.8)		15 (26.8)		.15		
	Atopic dermatitis	47 (16.8)		16 (17.8)		27 (20.1)		4 (7.1)		.09		
	Food allergies	24 (8.6)		11 (12.2)		7 (5.2)		6 (10.7)		.15		
**Triggers of wheezing episodes (nonexclusive), n (%)**												
	Respiratory infection	162 (57.9)		62 (68.9)^b^		84 (62.7)^b^		16 (28.6)^b,c^		<.001		
	Cold weather	87 (31.1)		35 (38.9)^b^		42 (31.3)		10 (17.9)^c^		.03		
	Exercise	57 (20.4)		24 (26.7)		25 (18.7)		8 (14.3)		.16		
	Inhalation exposure	34 (12.1)		17 (18.9)		13 (9.7)		4 (7.1)		.05		
	No risk factors	30 (10.7)		10 (11.1)		15 (11.2)		5 (8.9)		.89		
**Seasonality of respiratory catarrhal symptoms (nonexclusive), n (%)**												
	Spring	88 (31.4)		35 (38.9)^b^		45 (33.6)^b^		8 (14.3)^c^		.006		
	Summer	13 (4.6)		5 (5.6)		6 (4.5)		2 (3.6)		.85		
	Autumn	66 (23.6)		21 (23.3)		39 (29.1)^b^		6 (10.7)^a^		.02		
	Winter	101 (36.1)		39 (43.3)^b^		51 (38.1)^b^		11 (19.6)^b,c^		.01		
**Indoor environmental factors (nonexclusive), n (%)**												
	Indoor condensation	71 (25.4)		22 (24.4)		36 (26.9)		13 (23.2)		.85		
	Humid air	42 (15.0)		14 (15.6)		21 (15.7)		7 (12.5)		.84		
	Musty smell	20 (7.1)		8 (8.9)		7 (5.2)		5 (8.9)		.49		
	Water leakage	50 (17.9)		16 (17.8)		29 (21.6)		5 (8.9)		.11		
	Sunlight exposure	269 (95.7)		88 (97.8)		127 (94.8)		54 (96.4)		.52		
	Indoor plants	110 (39.3)		32 (35.6)		56 (41.8)		22 (39.3)		.65		
	Indoor smoking	132 (47.1)		44 (48.9)		59 (44.0)		29 (51.8)		.57		
	Cockroach present	38 (13.6)		13 (14.4)		19 (14.2)		6 (10.7)		.78		
	Pets present	28 (10.0)		11 (12.2)		9 (6.7)		8 (14.3)		.20		
	Stuffed toys	134 (47.9)		40 (44.4)		68 (50.7)		26 (46.4)		.63		
**Household hygiene products usage (nonexclusive), n (%)**												
	Air purifier	28 (10.0)		9 (10.0)		14 (10.4)		5 (8.9)		.95		
	Humidifier	55 (19.6)		13 (14.4)		33 (24.6)		9 (16.1)		.13		
	Dehumidifier	5 (1.8)		0 (0)		4 (3.0)		1 (1.8)		.26		
	Vacuum cleaner	54 (19.3)		15 (16.7)		31 (23.1)		8 (14.3)		.28		
	Fruit detergent	65 (23.2)		18 (20.0)		32 (23.9)		15 (26.8)		.62		
	Bathroom cleaner	204 (72.9)		68 (75.6)		92 (68.7)		44 (78.6)		.29		
	Laundry disinfectant	81 (28.9)		23 (25.6)		41 (30.6)		17 (30.4)		.69		
	Alcohol spray	91 (32.5)		32 (35.6)		42 (31.3)		17 (30.4)		.75		
**Medication use (nonexclusive), n (%)**												
	Inhaled medications	147 (52.5)		65 (72.2)^a,b^		70 (52.2)^b,c^		12 (21.4)^b,c^		<.001		
	Leukotriene receptor antagonists	67 (23.9)		24 (26.7)		36 (26.9)		7 (12.5)		.08		
	Antiallergic medications	71 (25.4)		31 (34.4)^b^		35 (26.1)^b^		5 (8.9)^b,c^		.003		
	Allergen immunotherapy	5 (1.8)		3 (3.3)		2 (1.5)		0 (0)		.32		
	Biologics	0 (0)		0 (0)		0 (0)		0 (0)		—		
	No medication	34 (12.1)		10 (11.1)		16 (11.9)		8 (14.3)		0.85		
**ACT^d^ or C-ACT^e^ (n=139), n (%)**												
	Controlled (≥20)	108 (77.7)		54 (74.0)		45 (80.4)		9 (90.0)		.43		
	Uncontrolled (<20)	31 (22.3)		19 (26.0)		11 (19.6)		1 (10.0)				

^a^Adjusted *P* value <.05 compared with the suspected asthma group.

^b^Adjust *P* value <.05 compared with the excluded asthma group.

^c^Adjusted *P* value <.05 compared with the confirmed asthma group.

^d^ACT: Asthma Control Test.

^e^C-ACT: Childhood Asthma Control Test.

### Determinants of Diagnostic Outcomes

**Figure 5:**
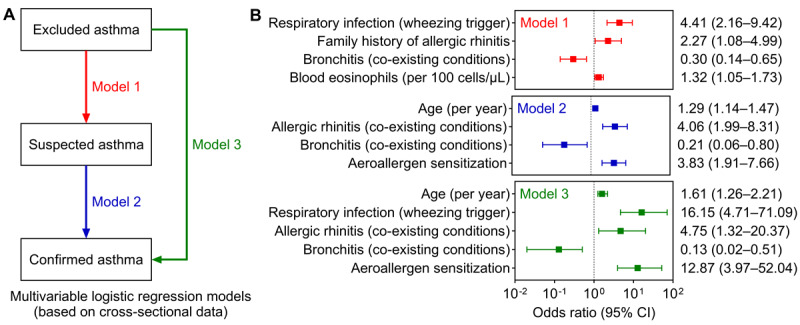
Determinants of diagnostic outcomes. (A) Schematic of the analytical models; (B) Results of the multivariable logistic regression analysis.

Three multivariable logistic regression models were constructed to identify factors associated with asthma diagnostic categories in this cross-sectional cohort (Figure 5A). In Model 1 (suspected vs excluded asthma), respiratory infection as a wheezing trigger (odds ratio [OR] 4.41, 95% CI 2.16-9.42, *P*<.001), family history of allergic rhinitis (OR 2.27, 95% CI 1.08-4.99, *P*=.03), and higher blood eosinophil counts (per 100 cells/μL, OR 1.32, 95% CI 1.05-1.73, *P*=.02) were significantly associated with suspected asthma (Figure 5B). In contrast, bronchitis was associated with reduced odds of suspected asthma (OR 0.30, 95% CI 0.14-0.65, *P*=.002).

Model 2 (confirmed vs suspected asthma) showed that confirmed asthma was associated with older age (per year, OR 1.29, 95% CI 1.14-1.47, *P*<.001), allergic rhinitis (OR 4.06, 95% CI 1.99-8.31, *P*<.001), and aeroallergen sensitization (OR 3.83, 95% CI 1.91-7.66, *P*<.001). Bronchitis remained negatively associated with confirmed asthma (OR 0.21, 95% CI 0.06-0.80, *P*=.02). These association patterns were consistent in Model 3 (confirmed vs excluded asthma). The robustness of these findings was confirmed through sensitivity analyses (Multimedia Appendix 2), supporting the clinical validity of the diagnostic stratification and highlighting specific features that distinguish asthma from alternative diagnoses.

## Discussion

### Principal Findings

This study describes the implementation of a prospective, longitudinal, and digitally enhanced real-world cohort framework that incorporated symptom-driven recruitment, standardized EMRs, ePROs, and residual biospecimen collection within a routine outpatient setting. Leveraging this framework, we enrolled a diagnostically heterogeneous population of children, with a focus on those younger than 6 years, and characterized their distinct clinical profiles according to categories of confirmed, suspected, or excluded asthma. These findings elucidate the broad spectrum of disease presentation in early life and highlight subgroups that have been historically challenging to systematically study, particularly in outpatient settings.

### Characteristics of Early-Life Asthma

The current data from the CRCA study provide an initial insight into the characteristics of young children presenting with asthmatic symptoms in an outpatient clinic. The high proportion of participants classified as suspected asthma (179/396, 45.2%), who were also the youngest at enrollment, reflects the diagnostic uncertainty common in early life. This finding aligns with a previous study using the term “reactive airway disease,” which reported a similarly high prevalence of 61.8% (249/403) in a comparable population [49]. The clinical profile of this group, which showed a lower prevalence of coexisting conditions such as allergic rhinitis and a lower rate of aeroallergen sensitization compared to children with confirmed asthma, yet also differed from the excluded asthma group in features such as lower rates of infectious conditions, suggesting that they may represent a distinct clinical group. This is particularly relevant because although the GINA 2025 criteria provide a definition for suspected asthma in young children, diagnosis remains primarily based on clinical history and lacks reliable biomarkers to support stratification [3]. Thus, the CRCA framework, with its integrated biospecimen repository, is well-positioned to offer future insights in this area. This observation warrants further longitudinal investigation to understand its implications for disease progression and management.

A notable finding was the discrepancy between physician diagnoses and parental perception of the child’s condition. The fact that a substantial proportion of parents did not concur with the physician’s diagnosis, regardless of whether asthma was confirmed or excluded, points to potential challenges in clinical communication and family education [50,51]. This discordance was most pronounced in the suspected asthma group (76/134, 56.7%), which is highly important since misalignment in perception in this particular group may lead to both under- and overtreatment, affecting disease management and medication use. This disconnect may be one factor influencing treatment adherence and outcomes in this age group.

A further focus of the CRCA is on early-life environmental exposures [52], which are currently captured largely through ePROs. Although no statistically significant differences were observed in the current group comparisons, this is likely due to insufficient sample size. Notably, the suspected asthma group showed the highest reported rate of household water leakage (29/134, 21.6%, *P*=.11) as well as the highest frequency of humidifier use (33/134, 24.6%, *P*=.13). These findings are of potentially important given that residential environments in Southwest China are already relatively humid, conditions that are closely associated with mold sensitization and asthma development in children [53-55]. Furthermore, CRCA’s emphasis on investigating household and personal disinfection and hygiene behaviors aligns with the theoretical frameworks of the hygiene hypothesis and the epithelial barrier hypothesis. As the sample size increases, this focus may support more detailed subgroup analyses in the future. In future phases, in addition to blood specimens, the CRCA plans to include the collection and biobanking of indoor dust samples to further enrich the repository. We also intend to expand our data collection to include maternal health and perinatal factors, thereby providing a more comprehensive assessment of early-life exposures.

To better characterize the distinguishing features of children across diagnostic categories, we constructed three regression models to simulate clinical diagnostic pathways. It must be noted, however, that these models were based on cross-sectional data and thus do not imply causality. We found that infectious triggers of wheezing, a family history of allergic rhinitis, and peripheral blood eosinophil count were the factors most supportive of a suspected asthma diagnosis; these three factors are not currently emphasized in the GINA 2025 guidelines [3]. Moving further along the diagnostic spectrum, older age, comorbid allergic rhinitis, and aeroallergen sensitization were identified as the factors most strongly associated with a confirmed asthma diagnosis. Furthermore, a coexistence of bronchitis was the primary factor arguing against asthma, which is consistent with GINA criteria. These findings align broadly with current clinical understanding [56], yet underscore the particular importance of comorbid allergic rhinitis and sensitization. Moreover, older age emerged as a strongly supportive factor for confirmed asthma, likely reflecting the gradual development of lung function and allergen sensitization throughout childhood. Although this may also be influenced by current diagnostic processes, it reinforces the notion that asthma diagnosis should be viewed through a developmental lens [49], potentially requiring a period of ongoing assessment rather than being considered a binary outcome determined in a single visit.

### Methodological Considerations Regarding the CRCA Framework

The CRCA framework demonstrates a practical operational model tailored to high-demand clinical environments like China’s, where implementing research poses substantial challenges [57]. Its shared-responsibility approach, which distributes data and biospecimen collection tasks between clinicians and participants, helps sustain operations within high-volume outpatient workflows. Rather than merely listing its components, the methodological value of CRCA lies in how these features collectively address specific gaps in real-world childhood asthma research. This is evident when contrasted with established international registries (Multimedia Appendix 3), which often prioritize confirmed diagnoses and may lack integrated biospecimen collection, but have substantially larger sample sizes than the CRCA [58-66].

The symptom-driven recruitment strategy, which is seldom used in conventional registries that typically require confirmed diagnoses, enables the enrollment of a heterogeneous patient population. This approach mirrors the diagnostic uncertainty common in early-life asthma and allows CRCA to capture disease features from the earliest symptomatic stages. Standard EMR systems are designed for clinical rather than research use, which often results in fragmented and incomplete data [67,68]. To address this inherent limitation, CRCA implemented standardized EMR templates and a preconsultation data collection step. This approach helps bridge the gap between clinical efficiency and research data quality in time-constrained environments. Furthermore, while ePROs are often optional or partially implemented in other registries, they constitute a core component of CRCA, which enhances its potential scalability and enables richer patient-centric data capture [32,33]. Similarly, biospecimen collection remains uncommon in large-scale registries due to clinical and logistical burdens; accordingly, CRCA’s residual sample-based model offers a pragmatic and scientifically valuable alternative [35-37,69]. This composition of interoperable, standardized modules suggests that portability to other clinical settings is possible.

It is important to position CRCA appropriately among existing studies. While large-scale, resource-intensive cohorts such as COPSAC have generated landmark findings through long-term follow-up and substantial infrastructure [9], CRCA operates within a different context and at a different developmental stage. Its value lies not in direct comparison, but in its seamless integration into routine clinical practice. This integration supports dynamic enrollment and scalable, real-world implementation, aligning with contemporary recommendations for integrating multiomics platforms and target trial methodologies to advance precision medicine under pragmatic conditions [28]. With further refinement, this clinic-embedded model may offer an adaptable framework for real-world evidence generation, especially in resource-constrained settings [39,70].

A challenge observed in this study was the initial follow-up rate (48/106, 45.3% among confirmed cases and 28/138, 20.3% among suspected cases), which reflects broader difficulties in pediatric asthma research. Contributing factors included the geographical dispersion of patients, gaps in parental awareness, and the inherent constraints of a regional referral center operating within a tiered health care system. These issues are not unique to CRCA, as comparable studies in the United States have reported similar follow-up rates (22.6%-55.0%) [41-43]. These realities underscore the importance of developing digital solutions for remote monitoring and self-management—a key future direction for CRCA [71,72]. In response, we have initiated the development of a digital medicine platform within the established CRCA WeChat public account. This platform is designed to integrate educational content, a mini-program for patient reporting, and an AI agent for interactive questions and answers, aiming to improve follow-up adherence and long-term engagement [73]. Additionally, the identification of an “off-day follow-up pattern” also suggests a need for more flexible scheduling options, such as night clinics, to improve adherence to review visits [74,75].

Looking forward, the “digitally enhanced” framework of CRCA could be positioned to support future innovation. We envision that the integrated data from EMRs and ePROs may serve as a basis for developing AI models that could aid in risk stratification. Similarly, the WeChat platform can be leveraged to extend care beyond the clinic, for instance, through remote monitoring, educational content, and AI-facilitated questions and answers. As these digital tools mature, CRCA could evolve into a testbed for evaluating scalable solutions that may improve asthma care in diverse clinical settings.

### Limitations

Several limitations should be considered when interpreting our findings. First, as a single-center study conducted in Southwest China, the CRCA cohort primarily comprises patients from Chongqing and neighboring provinces, which may limit the generalizability of our findings to other geographic or socioeconomic contexts. Second, the symptom-driven, outpatient-based recruitment strategy may introduce selection bias, as enrolled children likely represent a more symptomatic or health-seeking population than the general community. Third, the requirement for blood testing (serum sIgE or other tests), though aligned with the goal of residual biospecimen banking, may have excluded children with already confirmed asthma who were under routine treatment, further contributing to selection bias. Fourth, although the CRCA model successfully addressed recruitment challenges, follow-up rates remained suboptimal, particularly among younger children and those with uncertain diagnoses. This attrition may bias longitudinal analyses if loss to follow-up is associated with disease severity or remission. Finally, while we implemented standardized EMR templates and ePROs to enhance data quality, missingness in certain objective measures (eg, lung function, FeNO) was common and not imputed, which may affect the completeness of phenotypic characterization. This largely reflects age-specific clinical guidelines and efforts to minimize burden; future efforts will therefore prioritize novel, feasible biomarkers to strengthen objective profiling.

### Conclusions

The CRCA study demonstrates the feasibility of implementing a prospective, longitudinal, and digitally enhanced real-world cohort within routine pediatric outpatient practice. This initiative successfully captures the diagnostically complex population of children with asthmatic symptoms, with a particular focus on early-life presentations, and delineates a distinct clinical subgroup of children with suspected asthma. By integrating symptom-driven recruitment, standardized digital data collection, and biospecimen banking, the CRCA establishes a scalable platform for real-world evidence generation and may provide a valuable foundation for future research into asthma endotypes and personalized management approaches.

## Appendix

Multimedia Appendix 1 STROBE checklist.

Multimedia Appendix 2 Multivariable Logistic Regression and Sensitivity Analyses.

Multimedia Appendix 3 Comparison of the CRCA Cohort with Other Registry Studies.

## Abbreviations

ACT: Asthma Control Test
C-ACT: Childhood Asthma Control Test
CRCA: Clinical Registry of Childhood Asthma
EMR: electronic medical record
ePRO: electronic patient-reported outcome
FeNO: fractional exhaled nitric oxide
GINA: Global Initiative for Asthma
OR: odds ratio
PBMC: peripheral blood mononuclear cell
sIgE: specific IgE
STROBE: Strengthening the Reporting of Observational Studies in Epidemiology
