# Supplementary material for: The Clinical Registry of Childhood Asthma (CRCA) Elucidating Early-Life Asthma: Cross-Sectional Analysis of a Prospective, Longitudinal, and Digitally Enhanced Real-World Cohort
Source: J Med Internet Res. 2025 Oct 30;27:e78693. doi: 10.2196/78693 (PMC12616192; doi:10.2196/78693)
Supplement: Multimedia Appendix 1 [file jmir_v27i1e78693_app1.pdf]

Multimedia Appendix 1: STROBE Checklist

	Item No	Recommendation
**Title and abstract**	1	(*a*) Indicate the study's design with a commonly used term in the title or the abstract<br>**Title**: "A Prospective, Longitudinal and Digitally Enhanced Real-World Cohort"
		(*b*) Provide in the abstract an informative and balanced summary of what was done and what was found<br>**Abstract**: **Objectives**, **Methods**, **Results**, and **Conclusions** sections provide a complete summary.
**Introduction**		
Background/rationale	2	Explain the scientific background and rationale for the investigation being reported<br>**Introduction**, **paragraphs 1–4**
Objectives	3	State specific objectives, including any prespecified hypotheses<br>**Introduction**, **last paragraph**: "We aimed to characterize children across confirmed, suspected, and excluded asthma categories and identify factors associated with these diagnostic outcomes."
**Methods**		
Study design	4	Present key elements of study design early in the paper<br>**Methods → Study Design** (First paragraph)
Setting	5	Describe the setting, locations, and relevant dates, including periods of recruitment, exposure, follow-up, and data collection<br>**Methods → Study Design** (Launched March 7, 2024) & **Recruitment and Follow-up** (Follow-up every 4-6 months). **Results → Implementation and Feasibility of the CRCA Study** (Recruitment period: Mar 2024 - Aug 2025).
Participants	6	(*a*) *Cohort study*—Give the eligibility criteria, and the sources and methods of selection of participants. Describe methods of follow-up<br>*Case-control study*—Give the eligibility criteria, and the sources and methods of case ascertainment and control selection. Give the rationale for the choice of cases and controls<br>*Cross-sectional study*—Give the eligibility criteria, and the sources and methods of selection of participants<br>**Methods → Recruitment and Follow-up** (Eligibility criteria, recruitment source, follow-up schedule).
		(*b*) *Cohort study*—For matched studies, give matching criteria and number of exposed and unexposed<br>*Case-control study*—For matched studies, give matching criteria and the number of controls per case<br>**N/A** (Not a matched study)
Variables	7	Clearly define all outcomes, exposures, predictors, potential confounders, and effect modifiers. Give diagnostic criteria, if applicable<br>**Methods → Definitions of Asthma Diagnosis**; **Standard EMRs**; **ePROs**; **Statistical Analyses** (Variables included in models are listed).
Data sources/ measurement	8*	For each variable of interest, give sources of data and details of methods of assessment (measurement). Describe comparability of assessment methods if there is more than one group<br>**Methods → Standard EMRs**; **ePROs**; **Residual Biospecimens**; **Quality Control**

		**and Data Security**
Bias	9	Describe any efforts to address potential sources of bias<br>**Methods → Quality Control and Data Security** (Data verification, standardized protocols, biobanking standards).
Study size	10	Explain how the study size was arrived at<br>**N/A** (The study size was determined by the number of eligible participants enrolled during the study period, not by a formal sample size calculation).
Quantitative variables	11	Explain how quantitative variables were handled in the analyses. If applicable, describe which groupings were chosen and why<br>**Methods → Statistical Analyses** ("Categorical variables are presented as frequencies... continuous variables are shown as medians (IQRs)").
Statistical methods	12	(*a*) Describe all statistical methods, including those used to control for confounding<br>**Methods → Statistical Analyses**: "Three logistic regression models"... "forward stepwise likelihood ratio selection"... "age and sex retained in all models".
		(*b*) Describe any methods used to examine subgroups and interactions<br>**Methods → Statistical Analyses**: Subgroup analysis by diagnostic category is the primary analysis.
		(*c*) Explain how missing data were addressed<br>**Methods → Statistical Analyses**: "Missing data were not imputed"... reasons provided.
		(*d*) *Cohort study*—If applicable, explain how loss to follow-up was addressed<br>*Case-control study*—If applicable, explain how matching of cases and controls was addressed<br>*Cross-sectional study*—If applicable, describe analytical methods taking account of sampling strategy<br>**Methods → Recruitment and Follow-up**, **Results**, **Discussion**: Follow-up rates are reported and discussed as a limitation.
		(*e*) Describe any sensitivity analyses<br>**Methods → Statistical Analyses**: "sensitivity analyses to evaluate the stability of core associations" (Multimedia Appendix 2).
**Results**		
Participants	13*	(a) Report numbers of individuals at each stage of study—eg numbers potentially eligible, examined for eligibility, confirmed eligible, included in the study, completing follow-up, and analysed<br>**Results → Implementation...**: "2296 visits"... "396 eligible patients were enrolled"... follow-up numbers given.
		(b) Give reasons for non-participation at each stage<br>**Not explicitly stated** for recruitment. For follow-up, reasons are **discussed in the response to reviewers and inferred in Discussion** (geographical dispersion, parental awareness).
		(c) Consider use of a flow diagram<br>**Figure 3** includes a recruitment timeline and follow-up metrics.
Descriptive data	14*	(a) Give characteristics of study participants (eg demographic, clinical, social) and information on exposures and potential confounders<br>**Table 1** and **Table 2**.
		(b) Indicate number of participants with missing data for each variable of interest<br>**Table 1** footnotes provide denominators for variables with missing data (e.g., *n*=390 for sIgE).

		(c) *Cohort study*—Summarise follow-up time (eg, average and total amount)<br>**Results → Implementation...**: "Initial and second follow-up assessments were completed by 26.7%... and 43.3%".
Outcome data	15*	*Cohort study*—Report numbers of outcome events or summary measures over time<br>**Table 1** (Distribution of diagnostic groups: Confirmed 33.1%, Suspected 45.2%, Excluded 21.7%). **Figure 4**.
		Case-control study—Report numbers in each exposure category, or summary measures of exposure
		Cross-sectional study—Report numbers of outcome events or summary measures
Main results	16	(*a*) Give unadjusted estimates and, if applicable, confounder-adjusted estimates and their precision (eg, 95% confidence interval). Make clear which confounders were adjusted for and why they were included<br>**Results → Determinants of Diagnostic Outcomes** and **Figure 5B** (Adjusted ORs and 95% CIs). Univariate results are in **Table 1/2**.
		(*b*) Report category boundaries when continuous variables were categorized<br>**N/A** (Continuous variables were analyzed as continuous or reported as medians).
		(*c*) If relevant, consider translating estimates of relative risk into absolute risk for a meaningful time period<br>**N/A**
Other analyses	17	Report other analyses done—eg analyses of subgroups and interactions, and sensitivity analyses<br>**Results → Determinants of Diagnostic Outcomes**: "The robustness of these findings was confirmed through sensitivity analyses (Multimedia Appendix 2)".
**Discussion**		
Key results	18	Summarise key results with reference to study objectives<br>**Discussion → Principal Findings**
Limitations	19	Discuss limitations of the study, taking into account sources of potential bias or imprecision. Discuss both direction and magnitude of any potential bias<br>**Discussion → Limitations** (Single-center, selection bias, follow-up rate, missing data)
Interpretation	20	Give a cautious overall interpretation of results considering objectives, limitations, multiplicity of analyses, results from similar studies, and other relevant evidence<br>**Discussion → Characteristics of Early-Life Asthma** and **Methodological Considerations...**
Generalisability	21	Discuss the generalisability (external validity) of the study results<br>**Discussion → Limitations** (First limitation addresses generalizability).
**Other information**		
Funding	22	Give the source of funding and the role of the funders for the present study and, if applicable, for the original study on which the present article is based<br>**Acknowledgments**

*Give information separately for cases and controls in case-control studies and, if applicable, for exposed and unexposed groups in cohort and cross-sectional studies.
