# Supplementary material for: The Clinical Registry of Childhood Asthma (CRCA) Elucidating Early-Life Asthma: Cross-Sectional Analysis of a Prospective, Longitudinal, and Digitally Enhanced Real-World Cohort
Source: J Med Internet Res. 2025 Oct 30;27:e78693. doi: 10.2196/78693 (PMC12616192; doi:10.2196/78693)
Supplement: Multimedia Appendix 2 [file jmir_v27i1e78693_app2.pdf]

Multimedia Appendix 2: Multivariable Logistic Regression and Sensitivity Analyses

Model 1: Suspected asthma vs Excluded asthma

	Univariable analysis		Multivariable analysis 1 (P<.30, sensitivity analysis)		Multivariable analysis 2 (P<.20, sensitivity analysis)		Multivariable analysis 3 (P<.10, final model)	
**Variables**	**OR (95% CI)**	P	**OR (95% CI)**	P_{adj}	**OR (95% CI)**	P_{adj}	**OR (95% CI)**	P_{adj}
**Age, year (confounding variable)**	1.04 (0.93-1.17)	.49	1.11 (0.96-1.32)	.18	1.11 (0.96-1.32)	.18	1.11 (0.95-1.31)	.18
**Sex, male (confounding variable)**	1.35 (0.81-2.28)	.25	1.00 (0.48-2.05)	>.99	1.00 (0.48-2.05)	>.99	1.00 (0.49-2.04)	.99
**Preterm birth**	0.87 (0.31-2.45)	.80						
**Personal history of eczema**	2.16 (1.28-3.65)	.004	Excluded		Excluded		Excluded	
**Personal history of severe pneumonia**	0.87 (0.31-2.45)	.80						
**Co-existing Conditions**								
Allergic rhinitis	2.74 (1.17-6.46)	.02	Excluded		Excluded		Excluded	
Bronchitis	0.22 (0.12-0.40)	<.001	0.28 (0.13-0.62)	.002	0.28 (0.13-0.62)	.002	0.30 (0.14-0.65)	.002
Adenoid hypertrophy	0.83 (0.45-1.53)	.56						
Recurrent respiratory infections	0.62 (0.34-1.14)	.12	Excluded		Excluded		Not included	
Pneumonia	0.67 (0.25-1.82)	.43						
**Family history of related diseases**								
Asthma	2.84 (0.94-8.60)	.07	Excluded		Excluded		Excluded	
Allergic rhinitis	1.96 (0.99-3.89)	.05	2.23 (1.05-4.94)	.04	2.23 (1.05-4.94)	.04	2.27 (1.08-4.99)	.03
Atopic dermatitis	3.28 (1.09-9.87)	.03	Excluded		Excluded		Excluded	
Food allergies	0.46 (0.15-1.43)	.18	0.27 (0.07-1.00)	.05 (>.05)	0.27 (0.07-1.00)	.05 (>.05)	Not included	
**Triggers wheezing episodes**								
Respiratory infection	4.2 (2.13-8.27)	<.001	4.80 (2.32-10.51)	<.001	4.80 (2.32-10.51)	<.001	4.41 (2.16-9.42)	<.001
Cold weather	2.10 (0.97-4.56)	.06	Excluded		Excluded		Excluded	
Exercise	1.38 (0.58-3.27)	.47						
Inhalation exposure	1.40 (0.44-4.49)	.58						
**Seasonality of respiratory catarrhal symptoms**								

Spring	3.03 (1.32-6.96)	.009	Excluded		Excluded		Excluded	
Summer	1.27 (0.25-6.47)	.78						
Autumn	3.42 (1.36-8.63)	.009	Excluded		Excluded		Excluded	
Winter	2.51 (1.19-5.30)	.02	Excluded		Excluded		Excluded	
**Positive rate of serum allergen-specific IgE**								
Aeroallergens	1.53 (0.80-2.95)	.20	Excluded		Not included		Not included	
Food allergens	0.91 (0.51-1.64)	.76						
**Blood eosinophil count, 100 cells/µL**	1.26 (1.07-1.48)	.006	1.32 (1.05-1.72)	.02	1.32 (1.05-1.72)	.02	1.32 (1.05-1.73)	.02
**Indoor environmental factors**								
Indoor condensation	1.22 (0.59-2.52)	.60						
Humid air	1.30 (0.52-3.26)	.58						
Musty smell	0.56 (0.17-1.85)	.34						
Water leakage	2.82 (1.03-7.71)	.04	Excluded		Excluded		Excluded	
Sunlight exposure	0.67 (0.14-3.34)	.63						
Indoor plants	1.11 (0.59-2.10)	.75						
Indoor smoking	0.73 (0.39-1.37)	.33						
Cockroach present	1.38 (0.52-3.65)	.52						
Pets present	0.43 (0.16-1.19)	.10	Excluded		Excluded		Not included	
Stuffed toys	1.19 (0.64-2.22)	.59						
**Household hygiene products usage**								
Air purifier	1.19 (0.41-3.48)	.75						
Humidifier	1.71 (0.76-3.85)	.20	Excluded		Excluded		Not included	
Dehumidifier	1.69 (0.19-15.49)	.64						
Vacuum cleaner	1.81 (0.77-4.22)	.17	Excluded		Excluded		Not included	
Fruit detergent	0.86 (0.42-1.75)	.67						
Bathroom cleaner	0.60 (0.29-1.25)	.17	Excluded		Excluded		Not included	
Laundry disinfectant	1.01 (0.51-1.99)	.97						
Alcohol spray	1.05 (0.53-2.06)	.89						

Model 2: Confirmed asthma vs Suspected asthma

	Univariable analysis		Multivariable analysis 1 (*P*<.30, sensitivity analysis)		Multivariable analysis 2 (*P*<.20, sensitivity analysis)		Multivariable analysis 3 (*P*<.10, final model)	
**Variables**	**OR (95% CI)**	***P***	**OR (95% CI)**	P_{adj}	**OR (95% CI)**	P_{adj}	**OR (95% CI)**	P_{adj}
**Age, year (confounding variable)**	1.44 (1.30-1.60)	<.001	1.25 (1.11-1.42)		1.25 (1.11-1.42)	<.001	1.29 (1.14-1.47)	<.001
**Sex, male (confounding variable)**	1.08 (0.68-1.71)	.76	1.13 (0.56-2.30)		1.13 (0.56-2.30)	.74	1.11 (0.54-2.28)	.77
**Preterm birth**	1.40 (0.59-3.34)	.45						
**Personal history of eczema**	0.89 (0.56-1.41)	.61						
**Personal history of severe pneumonia**	1.13 (0.45-2.80)	.80						
**Co-existing Conditions**								
Allergic rhinitis	5.87 (3.53-9.74)	<.001	4.29 (2.11-8.89)		4.29 (2.11-8.89)	<.001	4.06 (1.99-8.31)	<.001
Bronchitis	0.54 (0.27-1.12)	.096	0.23 (0.06-0.78)		0.23 (0.06-0.78)	.02	0.21 (0.06-0.80)	.02
Adenoid hypertrophy	0.52 (0.27-0.97)	.04	Excluded		Excluded		Excluded	
Recurrent respiratory infections	0.33 (0.15-0.71)	.005	Excluded		Excluded		Excluded	
Pneumonia	0.53 (0.16-1.74)	.296	Excluded		Not included		Not included	
**Family history of related diseases**								
Asthma	1.07 (0.54-2.12)	.85						
Allergic rhinitis	0.81 (0.47-1.40)	.44						
Atopic dermatitis	0.86 (0.43-1.70)	.66						
Food allergies	2.53 (0.94-6.79)	.07	Excluded		Excluded		Excluded	
**Risk factors for wheezing episodes**								
Respiratory infection	1.32 (0.75-2.32)	.34						
Cold weather	1.39 (0.80-2.44)	.24	Excluded		Not included		Not included	
Exercise	1.59 (0.84-3.00)	.16	Excluded		Excluded		Not included	
Inhalation exposure	2.17 (1.00-4.72)	.05	Excluded		Excluded		Excluded	
**Seasonality of respiratory catarrhal symptoms**								
Spring	1.26 (0.72-2.19)	.42						
Summer	1.26 (0.37-4.24)	.72						

Autumn	0.74 (0.40-1.37)	.34						
Winter	1.25 (0.72-2.14)	.43						
**Positive rate of serum allergen-specific IgE**								
Aeroallergens	6.16 (3.73-10.19)	<.001	3.83 (1.94-7.71)	<.001	3.83 (1.94-7.71)	<.001	3.83 (1.91-7.66)	<.001
Food allergens	1.46 (0.89-2.41)	.14	Excluded		Excluded		Not included	
**Blood eosinophil count, 100 cells/μL**	1.25 (1.13-1.38)	<.001	Excluded		Excluded		Excluded	
**Indoor environmental factors**								
Indoor condensation	0.88 (0.48-1.63)	.69						
Humid air	0.99 (0.48-2.07)	.98						
Musty smell	1.77 (0.62-5.07)	.29	Excluded		Not included		Not included	
Water leakage	0.78 (0.40-1.54)	.48						
Sunlight exposure	2.43 (0.49-11.95)	.28	Excluded		Not included		Not included	
Indoor plants	0.77 (0.44-1.33)	.35						
Indoor smoking	1.22 (0.71-2.08)	.48						
Cockroach present	1.02 (0.48-2.19)	.96						
Pets present	1.93 (0.77-4.88)	.16	3.22 (1.10-9.54)	.03	3.22 (1.10-9.54)	.03	Not included	
Stuffed toys	0.78 (0.45-1.33)	.36						
**Household hygiene products usage**								
Air purifier	0.95 (0.39-2.30)	.91						
Humidifier	0.52 (0.26-1.05)	.07	Excluded		Excluded		Excluded	
Dehumidifier	-	-						
Vacuum cleaner	0.67 (0.34-1.32)	.24	Excluded		Not included		Not included	
Fruit detergent	0.80 (0.42-1.53)	.50						
Bathroom cleaner	1.41 (0.77-2.58)	.26	Excluded		Not included		Not included	
Laundry disinfectant	0.78 (0.43-1.42)	.41						
Alcohol spray	1.21 (0.69-2.13)	.51						

Model 3: Confirmed asthma vs Excluded asthma

	Univariable analysis		Multivariable analysis 1 (*P*<.30, sensitivity analysis)		Multivariable analysis 2 (*P*<.20, sensitivity analysis)		Multivariable analysis 3 (*P*<.10, final model)	
**Variables**	**OR (95% CI)**	***P***	**OR (95% CI)**	**P_{adj}**	**OR (95% CI)**	**P_{adj}**	**OR (95% CI)**	**P_{adj}**
**Age, year (confounding variable)**	1.62 (1.39-1.90)	<.001	1.61 (1.26-2.21)	<.001	1.61 (1.26-2.21)	<.001	1.61 (1.26-2.21)	<.001
**Sex, male (confounding variable)**	1.46 (0.84-2.53)	.18	0.35 (0.10-1.11)	.08	0.35 (0.10-1.11)	.08	0.35 (0.10-1.11)	.08
**Preterm birth**	1.22 (0.44-3.44)	.70						
**Personal history of eczema**	1.92 (1.11-3.33)	.02	Excluded		Excluded		Excluded	
**Personal history of severe pneumonia**	0.98 (0.34-2.87)	.98						
**Co-existing Conditions**								
Allergic rhinitis	16.09 (6.90-37.56)	<.001	4.75 (1.32-20.37)	.02	4.75 (1.32-20.37)	.02	4.75 (1.32-20.37)	.02
Bronchitis	0.12 (0.06-0.25)	<.001	0.13 (0.02-0.51)	.003	0.13 (0.02-0.51)	.003	0.13 (0.02-0.51)	.003
Adenoid hypertrophy	0.43 (0.21-0.88)	.02	Excluded		Excluded		Excluded	
Recurrent respiratory infections	0.20 (0.09-0.46)	<.001	Excluded		Excluded		Excluded	
Pneumonia	0.36 (0.10-1.25)	0.11	Excluded		Excluded		Not included	
**Family history of related diseases**								
Asthma	3.03 (0.96-9.52)	.06	Excluded		Excluded		Excluded	
Allergic rhinitis	1.58 (0.76-3.29)	.22	Excluded		Not included		Not included	
Atopic dermatitis	2.81 (0.89-8.89)	.08	Excluded		Excluded		Excluded	
Food allergies	1.16 (0.40-3.34)	.78						
**Risk factors for wheezing episodes**								
Respiratory infection	5.54 (2.66-11.51)	<.001	16.15 (4.71-71.09)	<.001	16.15 (4.71-71.09)	<.001	16.15 (4.71-71.09)	<.001
Cold weather	2.93 (1.31-6.54)	.009	Excluded		Excluded		Excluded	
Exercise	2.18 (0.90-5.27)	.08	Excluded		Excluded		Excluded	
Inhalation exposure	3.03 (0.96-9.52)	.06	Excluded		Excluded		Excluded	
**Seasonality of respiratory catarrhal symptoms**								
Spring	3.82 (1.62-9.02)	.002	Excluded		Excluded		Excluded	
Summer	1.59 (0.30-8.48)	.59						

Autumn	2.54 (0.95-6.74)	.06	Excluded		Excluded		Excluded	
Winter	3.13 (1.43-6.82)	.004	Excluded		Excluded		Excluded	
**Positive rate of serum allergen-specific IgE**								
Aeroallergens	9.44 (4.84-18.44)	<.001	12.87 (3.97-52.04)	<.001	12.87 (3.97-52.04)	<.001	12.87 (3.97-52.04)	<.001
Food allergens	1.33 (0.73-2.44)	.35						
**Blood eosinophil count, 100 cells/μL**	1.51 (1.28-1.79)	<.001	Excluded		Excluded		Excluded	
**Indoor environmental factors**								
Indoor condensation	1.07 (0.49-2.35)	.87						
Humid air	1.29 (0.49-3.42)	.61						
Musty smell	1.00 (0.31-3.21)	.99						
Water leakage	2.21 (0.76-6.40)	.15	Excluded		Excluded		Not included	
Sunlight exposure	1.63 (0.22-11.91)	.63						
Indoor plants	0.85 (0.43-1.70)	.65						
Indoor smoking	0.89 (0.46-1.74)	.73						
Cockroach present	1.41 (0.50-3.94)	.52						
Pets present	0.84 (0.31-2.22)	.72						
Stuffed toys	0.92 (0.47-1.80)	.82						
**Household hygiene products usage**								
Air purifier	1.13 (0.36-3.57)	.83						
Humidifier	0.88 (0.35-2.22)	.79						
Dehumidifier	-	-						
Vacuum cleaner	1.20 (0.47-3.05)	.70						
Fruit detergent	0.68 (0.31-1.50)	.34						
Bathroom cleaner	0.84 (0.38-1.87)	.68						
Laundry disinfectant	0.79 (0.38-1.65)	.53						
Alcohol spray	1.27 (0.62-2.59)	.52						

It should be noted that FEV1%Pred, FeNO, and ACT/C-ACT were not included due to small sample size. Furthermore, chronic cough was omitted from the multivariate analysis because it is frequently a component of the asthmatic phenotype rather than an independent predictor, and its inclusion could introduce collinearity.
