# Supplementary material for: The Clinical Registry of Childhood Asthma (CRCA) Elucidating Early-Life Asthma: Cross-Sectional Analysis of a Prospective, Longitudinal, and Digitally Enhanced Real-World Cohort
Source: J Med Internet Res. 2025 Oct 30;27:e78693. doi: 10.2196/78693 (PMC12616192; doi:10.2196/78693)
Supplement: Multimedia Appendix 3 [file jmir_v27i1e78693_app3.pdf]

Multimedia Appendix 3: Comparison of the CRCA Cohort with Other Registry Studies

Registry	Nation/ Region	Prospective/ Retrospective	Age	Symptom-driven recruitment	Electronic medical records (EMRs) / Electronic health records (EHRs)	Patient-reported outcomes (PROs) / Electronic PROs (ePROs)	Biological samples
International Severe Asthma Registry (ISAR) [1]	Global	Mixed	Adults	**No.** Eligible adults are those with severe asthma defined as requiring GINA Step 5 treatment or having uncontrolled asthma despite GINA Step 4 treatment.	**Yes.** It utilizes Electronic Data Capture (EDC) systems. In some countries, it is integrated into national EMR systems to avoid duplicate entry.	**Yes.** It collects PROs, including symptoms, quality of life, and asthma control.	**No.** The collection of biological samples is not mentioned.
Children's National Pediatric Asthma Registry [2]	United States	Retrospective	Children (0-17 years).	**No.** Eligible patients are children and adolescents who received care within the Children's National health system in the prior 24 months and have ≥1 ICD-10 diagnosis code containing the word "asthma."	**Yes.** Data is aggregated from three EHR systems (Cerner, eClinicalWorks, Greenway) used across the health network into a central Healthention Enterprise Data Warehouse.	**No.** The collection of PROs is not mentioned.	**No.** The collection of biological samples is not mentioned.
Primary Care Severe Asthma Registry (PCSAR) [3]	Canada	Mixed	Adults & Children (≥6 years)	**No.** Patients with suspected severe asthma are identified via EMR algorithms based on GINA/CTS criteria (e.g., high-dose medication use, exacerbations).	**Yes.** The registry is being built by extracting and analyzing data from the UTOPIAN primary care EMR database.	**No.** PROs are neither discussed nor collected.	**No.** The collection of biological samples is not mentioned.
Danish National Registry Study [4]	Denmark	Retrospective	Children (3-14 years)	**No.** All liveborn singletons in Denmark from 1995–1997 are included. Asthma cases are identified by ≥1 hospitalization	**Yes.** The study links data from multiple national registries (Birth, Patient, Prescription), which are populated with	**No.** PROs are not collected.	**No.** The collection of biological samples is not

				with an asthma diagnosis or ≥2 prescriptions for anti-asthmatic medications within 12 months (at age ≥3 years).	administrative and clinical data (similar to EMRs on a national scale).		part of this study.
Danish National Database for Asthma (DNDA) [5]	Denmark	Retrospective	Adults & Children (6-44 years)	**No.** Eligible patients have either a second purchase of asthma medication (ATC code R03) within a 2-year period or a hospital diagnosis of asthma (ICD-10: J45, J46).	**Yes.** It links national administrative registries (Patient, Prescription, Health Insurance). These registries contain data from healthcare encounters.	**No.** PROs are not collected.	**No.** The collection of biological samples is not part of this study.
Severe Paediatric Asthma Collaborative in Europe (SPACE) [6]	Europe	Prospective	Children (aged 6-17 years)	**No.** Eligible patients are children with severe asthma who have been managed at a specialized center for ≥6 months and meet strict clinical, spirometric, and high-dose treatment thresholds (per ERS/ATS guidelines).	**Yes.** Data is collected via a dedicated online case report form (CRF) platform. It may extract data from clinical records (which could include EMRs).	**Yes.** It collects the Paediatric Asthma Quality of Life Questionnaire (PAQLQ) score as a PROs.	**No.** The collection of biological samples is not part of this study.
Registry of Asthma Patients Initiating DUPIXENT (RAPID) [7]	Global	Prospective	Adults & Children (≥12 years)	**No.** Eligible patients have clinician-diagnosed asthma, initiate dupilumab for a primary indication of asthma (per country-specific labeling), and can comply with study procedures.	**Yes.** Data collection is based on standard clinical care.	**Yes.** It extensively uses PROs, including ACQ-6, MiniAQLQ, WPAI-Asthma, PALQ, PGA, and disease-specific questionnaires.	**No.** The collection of biological samples is not part of this study.
Mild/Moderate Asthma Network in Italy (MANI) [8]	Italy	Prospective	Adults	**No.** Eligible patients have a specialist diagnosis of mild-to-moderate asthma (per GINA 2020 guidelines) and are scheduled for a visit at a	**Yes.** Data collected via an electronic Case Report Form (eCRF) on the REDCap platform aligns with data "routinely reported in medical	**Yes.** Questionnaires are used to collect data on quality of life, patient engagement, disease awareness, asthma	**No.** The collection of biological samples is not part of this

				participating respiratory or allergy clinic.	records."	control, and environmental exposures.	study.
Health Economics of Allergic Diseases (HEAD) [9]	Europe	Retrospective	All ages	**No.** Eligible patients have a diagnosis of one or more target allergic diseases per specific criteria, received the diagnosis in 2018 or earlier, and had at least one visit for the disease at a participating specialist center in 2019.	**Yes.** Data collection is based on information "routinely reported in medical records" from participating specialist centers. An electronic Case Report Form (eCRF) on the REDCap platform is used.	**Yes.** PROs are collected, including data on missed work/school days, days lost by family members, and out-of-pocket costs.	**No.** The collection of biological samples is not part of this study.
Clinical Registry of Childhood Asthma (CRCA) [this study]	China	Prospective	Children (<18 years)	**Yes.** Eligible participants were children with persistent or recurrent cough and/or wheezing lasting ≥1 month, and who required blood tests	**Yes.** Standard EMRs are collected.	**Yes.** Structured ePROs are collected	**Yes.** Residual blood samples are collected at the present phase.

Note: Many countries maintain their own severe asthma registries; only the International Severe Asthma Registry is included here. It is important to note that this is not a systematic review or meta-analysis; therefore, the list presented is not exhaustive and may not include all existing national registries.

References:

1. ISAR Study Group. International Severe Asthma Registry: Mission Statement. Chest. 2020;157(4):805-814.
2. Shelef DQ, Badolato GM, Badh R, et al. Creation and validation of a citywide pediatric asthma registry for the District of Columbia. J Asthma. 2022;59(5):901-909.
3. D'Urzo KA, Tamari IE, Chapman KR, et al. Primary Care Severe Asthma Registry and Education Project (PCSAR-EDU): Phase 1 - an e-Delphi for registry definitions and indices of clinician behaviour. BMJ Open. 2022;12(3):e055958. Published 2022 Mar 24.
4. Chen Q, Chen J, Zhou Y, et al. Natural history and associated early life factors of childhood asthma: a population registry-based cohort study in Denmark. BMJ Open. 2021;11(11):e045728. Published 2021 Nov 25.
5. Backer V, Lykkegaard J, Bodtger U, Agertoft L, Korshoej L, Braüner EV. The Danish National Database for Asthma. Clin Epidemiol. 2016;8:601-606. Published 2016 Oct 25.
6. Liu NM, van Aalderen W, Carlsen KCL, et al. Severe Paediatric Asthma Collaborative in Europe (SPACE): protocol for a European registry. Breathe (Sheff).

2018;14(2):93-98.
7. Gall R, Jain N, Soong W, et al. Dupilumab-Treated Patients with Asthma in the Real World: The RAPID Global Registry. Adv Ther. 2023;40(3):1292-1298.
8. Braido F, Blasi F, Canonica GW, et al. Mild/Moderate Asthma Network in Italy (MANI): a long-term observational study. J Asthma. 2022;59(9):1908-1913.
9. Agache I, Torres M, Eguiluz-Gracia I, et al. Economic Impact of Allergic Diseases and Asthma-The HEAD Pan-European Registry. Allergy. 2025;80(6):1677-1701.
